# Maternal Fiber Dietary Intakes during Pregnancy and Infant Allergic Disease

**DOI:** 10.3390/nu11081767

**Published:** 2019-08-01

**Authors:** Rachelle A. Pretorius, Marie Bodinier, Susan L. Prescott, Debra J. Palmer

**Affiliations:** 1 School of Medicine, University of Western Australia, 35 Stirling Highway, Crawley 6009, Western Australia, Australia; 2 INRA Pays de la Loire, UR 1268 Biopolymers Interactions Assemblies, rue de la géraudière, BP 71627, Cedex 3, 44316 Nantes, France; 3 Telethon Kids Institute, University of Western Australia, 15 Hospital Ave, Nedlands 6009, Western Australia, Australia

**Keywords:** dietary fiber, allergic disease, pregnancy, infant, resistant starch

## Abstract

Maternal diet during pregnancy plays a likely role in infant immune development through both direct nutrient specific immunomodulatory effects and by modulating the composition and metabolic activity of the maternal gut microbiome. Dietary fibers, as major substrates for microbial fermentation, are of interest in this context. This is the first study to examine maternal intakes of different fiber sub-types and subsequent infant allergic disease. In an observational study of 639 mother–infant pairs (all infants had a family history of allergic disease) we examined maternal intakes of total fiber, soluble fiber, insoluble fiber, resistant starch, and prebiotic fiber, by a semi-quantitative food frequency questionnaire at 36–40 weeks’ gestation. Infants attended an allergy clinical assessment at 12 months of age, including skin prick testing to common allergens. Higher maternal dietary intakes of resistant starch were associated with reduced doctor diagnosed infant wheeze, adjusted odds ratio (aOR) 0.68 (95% CI 0.49, 0.95, *p* = 0.02). However, in contrast, higher maternal intakes of resistant starch were associated with higher risk of parent reported eczema aOR 1.27 (95% CI 1.09, 1.49, *p* < 0.01) and doctor diagnosed eczema aOR 1.19 (95% CI 1.01, 1.41, *p* = 0.04). In conclusion, maternal resistant starch consumption was differentially associated with infant phenotypes, with reduced risk of infant wheeze, but increased risk of eczema.

## 1. Introduction

Although family history remains a strong predictor of allergic disease [1,2,3,4], changes in the early environment are implicated in the dramatic increase in early-onset allergic disease, most notably the maternal diet and lifestyle during pregnancy [1,4,5,6]. The importance of the in-utero environment is further highlighted by evidence of emerging differences in immune function at birth in children who go on to develop allergic disease [6,7,8,9,10,11]. This underscores the need to identify the modifiable maternal and in-utero factors that influence early immune development [12]. Maternal nutrition, which is fundamental to all aspects of development, is of central interest in this context [13,14]. Traditional maternal dietary patterns rich in fish, fruit and vegetables, and specific nutrients (fiber, omega-3 fatty acids, zinc, selenium, and antioxidant vitamins) have been associated with reduced risk of allergic disease in offspring [13,14,15,16,17,18,19,20]. While many of these factors have direct immunomodulatory effects, there is growing evidence that immunoprotective benefits may also be mediated through favorable effects on the gut microbiome [12,21,22].

Of these, dietary fiber has been of central interest, with long-recognized multifaceted health benefits in reducing the long term risk of inflammatory diseases, such as cardiovascular disease [23], type 2 diabetes mellitus [24], and a range of malignancies [25]. Total dietary fiber is comprised of several sub-type components, including soluble fiber, insoluble fiber, prebiotics, and resistant starch [26,27]. Our knowledge of the role of these in immune development is still limited but likely to be acting, at least in part, by promoting a diverse microbiome [12,21,22], which has complex crosstalk with the immune system [28]. Short chain fatty acids (SCFA) and other metabolites produced when dietary fiber undergoes microbial fermentation [21,29] may influence host immune responses through several key pathways including: (1) promoting intestinal barrier integrity [30,31]; (2) anti-inflammatory effects via the activation of free fatty acid receptors, such as G-protein-coupled receptor (GPR) 43 and GPR41 on the surface of immune cells [20,32]; and (3) inhibition of Histone Deacetylases which regulate rapamycin (mTOR)-S6K pathways required for T cell differentiation into effector and regulatory cells [33,34]. Along with the mTOR pathways, SCFA also enhance the activation of STAT3, which is involved in the expression of cytokines (IL-10, IL-17, and IFN-γ) in T cells [33]. Collectively, these have the potential to alter the differential balance of Th1 versus Th2 immune differentiation [33,35].

The implications for infant immune development are less clear [29]. In humans, only a small number of studies have examined total dietary fiber intakes in relation to immune responses or allergic disease outcomes [20,32,36,37]. None of these studies have examined the influence of maternal intake of dietary fiber sub-types. Here, we have addressed this knowledge gap in a ‘high-risk’ of infant allergic disease (due to a history of allergic disease in at least one immediate family member) pregnancy cohort, to determine whether maternal dietary intakes of soluble fiber, insoluble fiber, prebiotics, and resistant starch, predict infant allergic disease outcomes at one year of age.

## 2. Materials and Methods

### 2.1. Study Population

All infant offspring studied in this cohort had a family history of allergic disease (asthma, allergic rhinitis, atopic dermatitis, and/or IgE-mediated food allergy) in at least one immediate family member. The maternal participants’ inclusion criteria were maternal age ≥18 years, non-smoker in pregnancy, gestation ≥36 weeks, and healthy pregnancy with no known complications (including immunodeficiency, pre-eclampsia, major congenital anomalies). The sample size aim of this cohort was to have >600 mother–infant pairs with infant allergy outcomes at 12 months of age, which was based on previous cohorts which have examined other aspects of maternal diet in pregnancy and infant allergic disease outcomes, and taking into account our enriched “at-risk” of allergic disease due to family history cohort approach [15,16]. The cohort was set-up to examine multiple aspects of maternal diet and lifestyle in addition to the focus of this analysis on dietary fiber. This study was conducted in Perth, Western Australia, and approved by the Princess Margaret Hospital Human Research Ethics Committee (HREC approval number 1942EP). All participants provided written informed consent.

### 2.2. Maternal Dietary Assessment

During the recruitment visit at 36–40 weeks gestation maternal baseline data were collected, including history of allergic disease, education, ethnicity, parity, and pet ownership (cat, dog, or both), and a maternal diet semi-quantitative food frequency questionnaire (SQFFQ) was administered [38,39]. The SQFFQ was developed and analyzed by The Cancer Epidemiology & Intelligence Division, Cancer Council Victoria, Australia, and reported frequency of 101 individual foods, mixed foods, and beverages that were regularly eaten over the previous month (32–36 weeks of gestation). The SQFFQ was used to calculate the total dietary fiber intake measured in grams per day (g/day) for each woman participant. The amount of the total dietary fiber in each single food item was then calculated as total dietary fiber (gram) in 100 g of each food item (g/100 g) and the dietary fiber sub-types (insoluble fiber, soluble fiber, prebiotics, and resistant starch) for each single food item was calculated. For soluble fiber and insoluble fiber three sources were used: (1) latest dietary fiber values published by the Australian Bureau of Statistics (ABS) data (AUSNUT 2011-13) from Food Standards Australia New Zealand (FSANZ) (FSANZ, 2019) [40,41], (2) food industry provided dietary fiber composition data published by FSANZ (FSANZ, 2013), and (3) the published dietary fiber data by the FSANZ in “Composition of Foods”, Australia, 1989 [42]. For resistant starch, the maternal dietary intakes from each food item (g/100 g) was calculated using data from the food industry provided dietary fiber composition data (FSANZ, 2013) and the FSANZ publication “Composition of Foods”, Australia, 1989 [42]. The Australian online nutritional analysis software programs FoodWorks [43] and Foodzone [44] were also used to cross check and complete the data. For the prebiotic dietary fiber content in foods (g/100 g), values determined, and published by Muir et al. 2007, Muir et al. 2009, Van Loo et al. 1995, and Biesiekierski et al. 2011 were used [45,46,47,48]. Total dietary fiber (g/day) from fruit, vegetables, green vegetables, grain, and starch and gluten rich food was also calculated. To control for under or over reporting of maternal dietary intakes, women who reported unrealistic energy intake estimates of below 4500 kJ or above 20,000 kJ per day were excluded, as per methodology in previous dietary analysis studies of women during pregnancy [49,50].

### 2.3. Infant Clinical Outcomes Assessment

When the infants were 12 months of age, an allergy clinical assessment appointment was conducted. A parent was asked if the infant experienced any typical eczema skin lesions during the last 12 months (parent reported eczema), if the eczema was diagnosed by a medical doctor (doctor diagnosed eczema), and if the eczema skin lesions were responsive to topical steroid treatment prescribed by a medical doctor (steroid treated eczema). The parent was also asked about the child’s history of wheeze symptoms during infancy (parent reported wheeze), and if any wheeze symptoms had been diagnosed by a medical doctor (doctor diagnosed wheeze). IgE-mediated food allergy was based on history of immediate IgE-mediated symptoms (within 60 min of food ingestion) including angioedema, urticaria, cough, wheeze, stridor, vomiting, diarrhea, and/or cardiovascular symptoms, and allergen sensitization to the same food detected by positive skin prick test (SPT) at the 12 month of age visit. SPT was conducted to detect allergen sensitization to common Australian food and environmental allergens (cow’s milk, egg, peanut, cashew nut, wheat, rye grass, house dust mite, and cat; Hollister-Stier Laboratories, Spokane, WA, USA), as well as histamine as a positive control. A response was considered positive if the mean of the horizontal and perpendicular wheal diameter was 3 mm or greater in size than the mean wheal of the negative control site at 15 min. Sensitization was defined as a positive skin prick test result to at least one of the allergens assessed. Each child’s clinical allergy assessment results were confirmed by the research physician. Infant birth details (including delivery mode, gestational weight, gestational age, and infant gender) were also collected.

### 2.4. Statistical Analysis

All statistical analyses were performed using SPSS software (version 21.0 for Macintosh, SPSS Inc., Chicago, IL, USA). We used a binary (present or absent) classification to separately classify each of the allergic disease clinical outcomes. Total dietary fiber, soluble dietary fiber, insoluble dietary fiber, prebiotic fiber, resistant starch, as well as total dietary fiber from fruit, vegetables, green vegetables, grains, and gluten rich food were not normally distributed and were analyzed using non-parametric tests and results expressed as median and interquartile ranges (IQR). Univariate analyses were performed to determine the non-nutrient characteristics (demographics and environmental factors) associated with the clinical outcomes by using logistic regression. The final multivariable model included a comprehensive set of environmental and demographic risk factors which Nurmatov et al. (2012) had identified as confounders when studying the association between diet and childhood allergies [51]. These risk factors included: (1) maternal age, (2) maternal education, (3) ethnicity, (4) child’s gender, (5) child’s birth weight, (6) child’s gestational age at birth, (7) pet ownership, (8) maternal parity and (9) delivery mode. The logistic regression analysis was then applied to estimate the crude (unadjusted) odds ratio (OR) and their 95% confidence interval (CI) interval for the eczema, wheeze, food allergy, and allergen sensitization infant outcomes. After the unadjusted association of maternal dietary fiber sub-types and allergic disease was examined, we calculated an adjusted model that included the above mentioned environmental and demographic risk factors. Differences were regarded as significant at the level *p* < 0.05.

## 3. Results

### 3.1. Study Population Characteristics

A total of 930 mothers were initially recruited into this observational study. Four women were excluded from this current analysis due to missing baseline demographic data, 142 women were excluded due to missing SQFFQ data, and 22 women were excluded due to reported energy consumption data falling out of the pre-defined cut-offs. In total, 639 mother–infant pairs had infant allergy clinical outcome data available from the follow-up visit at 12 months of age, as 123 infants did not complete the follow-up assessments. The study population maternal and infant demographic data are summarized in Table 1.

### 3.2. Maternal Dietary Intake During Late Pregnancy

The maternal study population consumed a median (interquartile range (IQR)) energy intake of 8082 (6612–9845) kJ/day and 208 (156–268) g/day of total dietary carbohydrate intake. The participants had a total dietary fiber intake median of 23.8 (19.0–29.0) g/day. Median dietary fiber sub-type intakes of the participants are shown on Table 2.

### 3.3. Infant Allergic Disease Outcomes

**Figure 1:**
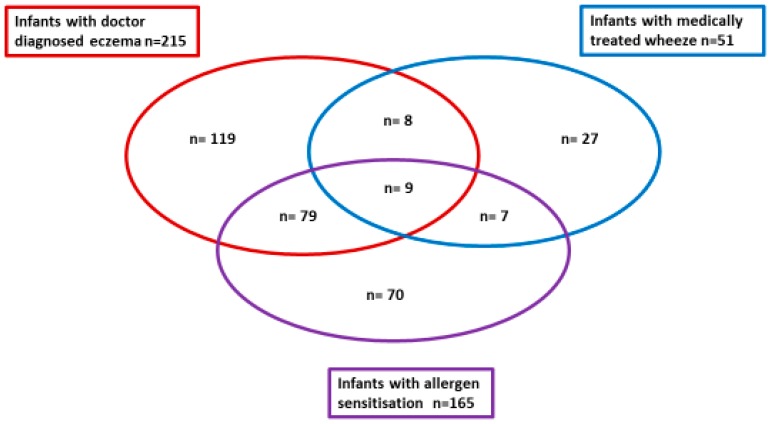
Infant clinical phenotype clusters of doctor diagnosed eczema, medically treated wheeze, and allergen sensitization outcomes during infancy. There were only 17 infants in total with both doctor diagnosed eczema and medically treated wheeze clinical phenotypes.

In this high-risk cohort (family history of allergic disease) during the first 12 months of life, 288/639 (45.1%) infants had parent reported eczema, 215/635 (33.9%) had doctor diagnosed eczema, and 166/630 (26.3%) had steroid treated eczema. During the first 12 months of life, 166/638 (26.0%) infants had parent reported wheeze, and 51/629 (8.1%) had doctor diagnosed wheeze. Overall 165/629 (26.2%) infants were sensitized to one or more allergens on SPT, with 129 infants to one or more food allergens only, 35 to one or more aero-allergens only, and only one infant had co-existing food and aero-allergen sensitization at one year of age. Diagnosis of IgE-mediated food allergy to one or more foods occurred in 91/632 (14.4%) infants. Figure 1 illustrates the infant clinical phenotype clusters of doctor diagnosed eczema, medically treated wheeze and allergen sensitization outcomes during infancy. In total, 249/639 (39.0%) of the participating infants had either doctor diagnosed eczema and/or medically treated wheeze, however as illustrated in Figure 1, there was limited overlap with only 17/639 (2.7%) infants with both clinical phenotypes. In this cohort, there were no statistical differences between infants who were delivered by vaginal birth compared to caesarean birth for any of the infant allergy outcomes (wheeze, eczema, IgE-mediated food allergy, or allergen sensitization). For those infants with medically treated wheeze more (*p* < 0.01) were boys (70.6%) than girls (29.4%), and for allergen sensitization again more (*p* = 0.03) were boys (59.4%) than girls (40.6%), but there were no gender differences for the eczema or IgE-mediated food allergy outcomes. Caucasian infants had less allergen sensitization (*p* < 0.01) and less IgE-mediated food allergy (*p* = 0.01) compared to infants from other ethnic backgrounds. Pet ownership was protective for reduced IgE-mediated food allergy (*p* = 0.02), but not for other infant allergy outcomes.

### 3.4. Maternal Dietary Intakes and Infant Allergic Disease Outcomes

Table 3 summarizes the median (IQR) maternal dietary intakes of energy, carbohydrate, total fiber, fiber sub-types, and some major fiber food sources, comparing infants with or without wheeze and eczema clinical outcomes. Mothers of infants with parent reported wheeze consumed less soluble fiber (*p* = 0.02) and less fruit fiber sources (*p* = 0.004) during late pregnancy. Similarly, mothers of infants with doctor diagnosed wheeze had lower maternal dietary intakes of resistant starch (*p* = 0.02). In contrast, higher maternal intakes of resistant starch were associated with increased doctor diagnosed eczema (*p* = 0.01). Furthermore, infant doctor diagnosed eczema was also associated with higher maternal intakes of carbohydrate (*p* = 0.01), total dietary fiber (*p* = 0.04), soluble fiber (*p* = 0.01), dietary fiber in grains (*p* = 0.02), fiber from gluten rich foods (*p* = 0.01), and fiber from green vegetables (*p* = 0.04) in late pregnancy. Steroid treated eczema was associated with higher maternal carbohydrate (*p* = 0.03) and soluble fiber (*p* = 0.03) dietary intakes. In this cohort, there were no associations found between maternal dietary intakes of the nutrients and foods examined and infant allergen sensitization status or IgE-mediated food allergy outcomes (Table 4).

### 3.5. Adjusted Model Results of Maternal Dietary Fiber Intakes and Infant Clinical Outcomes

After adjustment for potential confounders, the relationship with maternal dietary intakes remained significant for the association between higher maternal resistant starch intakes and lower risk of doctor diagnosed wheeze, adjusted odds ratio (aOR) 0.68 (95% CI 0.49, 0.95, *p* = 0.02) (Table 5). Again, after adjustment for potential confounders, and in contrast to the wheeze outcome results, higher maternal intakes of resistant starch were associated with higher risk of parent reported eczema aOR 1.27 (95% CI 1.09, 1.49, *p* < 0.01) and doctor diagnosed eczema aOR 1.19 (95% CI 1.01, 1.41, *p* = 0.04). Using multinominal logistic regression, higher maternal resistant starch dietary intakes late pregnancy were identified to be specifically associated with infant doctor diagnosed eczema without allergen sensitization aOR 1.29 (95% CI 1.06, 1.57, *p* = 0.01). Higher maternal intakes of fiber from green vegetables were also associated with higher risk of infant doctor diagnosed eczema aOR 1.32 (95% CI 1.06, 1.64, *p* = 0.01), and again this was specifically associated with eczematous infants without allergen sensitization aOR 1.36 (95% CI 1.04, 1.79, *p* = 0.03).

## 4. Discussion

Dietary fiber is a major determinant of the characteristics and composition of the human gut microbiome and associated fermentation patterns. Fiber sub-types (soluble fiber, insoluble fiber, resistant starch, and prebiotics) have different structural compositions, characteristics, and fermentation patterns that contribute to different metabolites, with resulting implications for local and systemic immune function [52,53,54]. Animal studies suggest maternal fiber intakes in pregnancy mediate effects on fetal lung development, allergic airway inflammation, and the developing immune regulatory pathways, and that this is mediated through SCFA metabolites by inhibiting histone deacetylases (HDACs) leading to transcription of Foxp3 [20,55]. Here, consistent with another human observational study by Thorburn et al. [20], we observed a protective relationship between maternal fiber (resistant starch) dietary intakes and reduced infant wheeze. Although wheeze symptoms can pre-exist in children later diagnosed with asthma, wheeze during infancy is nonspecific and represents a mixed group of phenotypes, including infants who may have an emerging allergic phenotype, but many are transient wheezers without reactive airways disease. Aeroallergen sensitization is also uncommon at this age, as detected in only 36/639 (5.6%) infants in this cohort, making it harder to delineate these phenotypes. A recent study by Michael et al. [56] provides possible mechanistic insight on these observed protective effects on infant wheeze outcomes, as they treated lung epithelial cells with prebiotics and found facilitation of wound repair in these human bronchial epithelial cells involving mannose receptors.

At present, we have limited knowledge regarding any effects on the developing skin barrier function and cutaneous disease. One animal study has suggested that prebiotics in pregnancy reduce eczema-like inflammation in offspring [57], but the mechanisms are not clear and there are no human studies. Here, we found an unexpected association between maternal dietary fiber intakes and infant eczema outcomes. Rather than co-associate with the wheezing phenotype, we instead found that higher consumption of several fiber types, most notably resistant starch, was associated with higher risk for eczema. In infancy, eczema is a more specific indicator of an emerging allergic phenotype [58] than wheeze, and our findings are not consistent with the hypothesis that higher maternal dietary fiber intakes protect from an infant allergic phenotype. Interestingly though, it was specifically the sub-group of infants with doctor diagnosed eczema and without allergen sensitization whose mothers had consumed higher resistant starch intakes.

This further highlights the need to understand the different pathways conferring both risk and protection from inflammation in different organ systems, before and after birth. There is growing speculation that organ-specific inflammation may have its origins in barrier dysregulation [59]. The local factors affecting the barrier function in the skin and respiratory mucosa are likely to be different. Similarly, the relative contribution to pre- and post-natal factors is also likely to vary. While there are postnatal associations between gut barrier integrity and skin barrier function in infants, the role of antenatal dietary factors and the maternal microbiome and metabolome is unclear. Clinical trials may also be helpful in examining the relationship between antenatal prebiotic intakes and skin integrity in the immediate postnatal period—as a measure of antenatal conditioning—as well as subsequent eczema outcomes.

A limitation of this dietary analysis was that we were unable to further sub-divide maternal resistant starch dietary intakes into the four sub-types of resistant starch (RS1, RS2, RS3, RS4), which have different structural composition and functional properties. The effect of these on infant immune function and development, with potential varied impact on the allergic phenotype, requires further investigation.

In our cohort, we did find family pet ownership, infant gender, and ethnicity differences for the infant allergy outcomes, which is consistent with other observational studies, including the large (*n* > 5000) general population HealthNuts Study from Victoria, Australia [2,5]. Nurmatov et al. (2012) [51] determined a comprehensive set of confounders (maternal age, maternal education, ethnicity, child’s gender, child’s gestational weight, child’s gestational age, pet ownership, maternal parity, and delivery mode) that should be included in epidemiological studies investigating the role of diet in the development of childhood allergic disease, hence these confounders were used in the adjusted model. The unadjusted odds ratios and adjusted odds ratios (aOR) are all presented in Table 5, but adjusting with the nine confounder variables did not change the significant infant allergy outcomes for maternal consumption of resistant starch, which is our major significant association finding in this pregnancy cohort.

Other maternal and infant dietary factors and lifestyle exposures can also influence the development of infant allergy outcomes. These may include maternal consumption of other nutrients and dietary factors, infant breast milk and/or infant formula feeding practices, as well as the timing of introduction of various allergenic solid foods (for example egg and peanut). In particular, the human milk oligosaccharide profile of breast milk can play an important role on the establishment of the infant gut microbiome. However, in this cohort, a limitation to acknowledge is that we did not collect detailed infant feeding data (especially exclusivity of breastfeeding and accurate infant formula use) between birth and 12 months of age, nor did we collect maternal or infant stool samples. Our aim of this investigation was to address a particular knowledge gap which was to focus on the question of whether maternal dietary intakes of various fiber sub-types (soluble fiber, insoluble fiber, prebiotics, and resistant starch) can be associated with infant allergic disease outcomes. This observational cohort analysis was conducted in the lead up to our current randomized controlled trials investigating maternal dietary prebiotic supplementation during pregnancy.

In this study, we did not see any associations with maternal prebiotic fiber sub-type dietary intake and the infant outcomes assessed. This may reflect the small quantities of prebiotics consumed in our study population. While the average total dietary fiber intake was 23.8 g/day and resistant starch intake was 2.8 g/day (both similar to consumption levels previously reported in other Australian studies [40]), the average prebiotic fiber intake was only 1.4 g/day. This prebiotic level of consumption is lower than a typical Mediterranean-style diet of 3–11 g/day of prebiotic fiber, and is more akin to the Western-style diet reported in the United States of 1–4g prebiotics per day [48]. Studies showing clinical or immunomodulation effects of prebiotic fiber intakes and associated increased SCFA production have had higher reported intakes or specific supplementation with prebiotics [60,61,62,63,64] and these studies have been limited to the postnatal period. This adds to the need for clinical trials in pregnancy to better characterize the effects of dietary fiber sub-types on the maternal microbiome, and associated effects on the metabolome (of mother and infant) and developing infant immune function. Investigation of any effects on breast milk composition may also provide another pathway through which maternal dietary fiber and prebiotics specifically may influence infant immune programming and allergy risk. To directly address this, we are currently undertaking two randomized controlled trials (in Australia ACTRN12615001075572 and in Europe NCT03183440) that will assess the effects of maternal prebiotic fiber supplementation during pregnancy on infant allergic disease outcomes.

## 5. Conclusions

This is the first observational analysis of different maternal dietary fiber sub-type intakes in relation to infant allergic disease outcomes. While it confirms some trends seen in other studies in relation to the protection against infant wheeze, it raises further questions about the role in other aspects of the infant allergic phenotype development, namely eczema. This further underscores the need for interventional studies and the anticipated importance of the randomized controlled trials that are currently underway to examine the potential of maternal prebiotics consumption in allergy prevention. Samples collected during these studies will also provide opportunities to examine mechanistic pathways that will enhance our understanding in this field.

## Figures and Tables

**Table 1:** Study population (*n* = 639) maternal and infant demographical characteristics.

Maternal and Infant Characteristics	
Maternal age (years) ^a^	32 (30–36)
Maternal allergic disease history ^b^	579 (90.6%)
Maternal ethnicity (Caucasian) ^b^	563 (91.1%)
Education level (further education post high school) ^b^	474 (74.6%)
Multivitamin consumption in pregnancy ^b^	525 (86.8%)
Pet ownership in pregnancy (cat, dog, or both) ^b^	382 (59.8%)
First born child ^b^	306 (47.9%)
Vaginal birth ^b^	416 (66.5%)
Child gender (male) ^b^	333 (52.1%)
Infant gestational age at birth (weeks) ^a^	39 (39–40)
Infant weight at birth (g) ^c^	3496 (±431)

^a^ median (IQR); ^b^ number (percentage); ^c^ mean ± SD.

**Table 2:** Study population (*n* = 639) maternal dietary intakes during late pregnancy.

Dietary Intake Variables	Median (IQR)
Energy (kJ/day)	8082 (6612–9845)
Carbohydrate (g/day)	207.7 (156.3–268.2)
Total dietary fiber (g/day)	23.8 (19.0–29.0)
Soluble dietary fiber (g/day)	5.6 (4.4–6.9)
Insoluble dietary fiber (g/day)	6.7 (5.0–8.6)
Prebiotic fiber (g/day)	1.4 (1.1–2.0)
Resistant starch (g/day)	2.8 (2.1–3.5)
Total fiber from grains (g/day)	9.2 (7.0–11.9)
Total fiber from fruit (g/day)	3.5 (2.2–5.2)
Total fiber from vegetables (g/day)	3.5 (2.5–4.8)
Total fiber from green vegetables (g/day)	1.1 (0.7–1.6)

**Table 3:** Maternal dietary intakes (median and interquartile ranges (IQR)) during late pregnancy and infant wheeze and eczema outcomes.

	Parent Reported Wheeze		Doctor Diagnosed Wheeze		Parent Reported Eczema		Doctor Diagnosed Eczema		Steroid Treated Eczema	
**Maternal dietary intakes**	Yes	No	Yes	No	Yes	No	Yes	No	Yes	No
	166/638 (26%)	472/638 (74%)	51/629 (8%)	578/629 (92%)	288/639 (45%)	351/639 (55%)	215/635 (34%)	420/635 (66%)	166/630 (26%)	464/630 (74%)
**Energy (kJ/day)**	7846	8135	7897	8093	8164	7900	8104	7974	8190	7926
	(6294–9836)	(6771–9869)	(5770–9858)	(6758–9864)	(6894–9861)	(6358–9835)	(6994–9861)	(6390–9840)	(6849–10211)	(6507–9731)
***p*-value**	0.07		0.15		0.18		0.22		0.15	
**Carbohydrate**	200.43	208.99	193.63	209.43	215.32	202.24	216.34	203.27	215.39	205.41
**(g/day)**	(151.49–254.37)	(159.62–272.33)	(143.04–257.06)	(156.82–270.76)	(168.25–273.97)	(150.84–261.87)	(173.39–274.20)	(149.37–262.90)	(174.41–274.74)	(150.11–262.19)
***p*-value**	0.34		0.28		0.05		**0.01**		**0.03**	
**Total fiber (g/day)**	23.04	24.14	22.75	24.01	24.58	22.68	25.01	23.1	25.01	23.27
	(17.71–27.65)	(19.32–29.56)	(17.50–25.78)	(19.14–29.36)	20.25–29.03)	(17.96–29.04)	(20.29–28.68)	(18.19–29.20)	(20.23–28.63)	(18.28–29.07)
***p*-value**	0.81		0.1		**0.01**		**0.04**		0.81	
**Soluble fiber**	5.23	5.73	5.01	5.66	5.65	5.54	5.71	5.5	5.71	5.52
**(g/day)**	(4.29–6.73)	(4.59–7.09)	(4.20–6.70)	(4.44–6.99)	(4.43–6.94)	(4.38–6.94)	(4.47–7.08)	(4.37–6.89)	(4.45–7.12)	(4.38–6.81)
***p*-value**	**0.02**		0.08		0.8		**0.01**		**0.03**	
**Insoluble fiber**	6.35	6.77	6.68	6.71	6.36	6.76	6.85	6.65	6.71	6.67
**(g/day)**	(4.98–8.43)	(5.07–8.73)	(5.20–9.37)	(5.02–8.59)	(4.92–8.44)	(5.23–8.98)	(5.09–8.61)	(5.00–8.63)	(5.04–8.62)	(5.00–8.61)
***p*-value**	0.15		0.65		0.12		0.75		0.9	
**Prebiotic fiber**	1.45	1.44	1.38	1.44	1.52	1.39	1.52	1.42	1.51	1.43
**(g/day)**	(1.02–1.96)	(1.10–2.95)	(0.97–1.98)	1.09–1.95)	(1.06–1.98)	(1.09–1.92)	(1.06–1.99)	(1.08–1.94)	(1.06–21.97)	(1.08–1.93)
***p*-value**	0.84		0.6		0.46		0.53		0.93	
**Resistant starch**	2.58	2.84	2.46	2.84	2.93	2.64	2.99	2.67	2.97	2.71
**(g/day)**	(2.04–3.44)	(2.16–3.53)	(2.02–3.05)	(2.13–3.54)	(2.34–3.59)	(2.03–3.41)	(2.34–3.56)	(2.06–3.47)	(2.26–3.54)	(2.10–3.50)
***p*-value**	0.18		**0.02**		**<0.01**		**0.01**		0.11	
**Total fiber, grains (g/day)**	9.25	9.36	7.64	9.33	9.43	9.09	9.58	8.99	9.55	9.14
	(6.52–11.09)	(7.06–11.85)	(5.76–12.23)	(7.0–11.8)	(7.4–11.8)	(6.73–12.05)	(7.52–12.04)	(6.69–11.62)	(7.47–11.92)	(6.73–11.70)
***p*-value**	0.21		0.12		0.2		**0.02**		0.12	
**Total fiber, gluten**	7.31	7.81	6.41	7.73	8.11	7.2	8.1	7.42	7.91	7.41
**rich foods (g/day)**	(5.10–10.31)	(5.50–10.40)	(4.40–10.90)	(5.51–10.32)	(5.71–10.50)	(5.10–10.21)	(5.91–10.90)	(5.10–10.21)	(5.61–10.80)	(5.10–10.31)
***p*-value**	0.35		0.14		**0.02**		**0.01**		0.09	
**Total fiber, green**	1.18	1.08	1.13	1.11	1.11	1.11	1.19	1.09	1.17	1.09
**vegetables (g/day)**	(0.8–1.8)	(0.70–1.58)	(0.84–1.76)	(0.70–1.62)	(0.75–1.70)	(0.65–1.55)	(0.77–1.77)	(0.68–1.55)	(0.79–1.62)	(0.68–1.63)
***p*-value**	0.31		0.57		0.25		**0.04**		0.19	
**Total fiber, fruit**	3.14	3.69	3.33	3.35	3.61	3.51	3.61	3.52	3.86	3.47
**(g/day)**	(1.9–4.8)	(2.37–5.44)	(1.94–4.91)	(2.26–5.23)	(2.20–5.22)	(2.25–5.14)	(2.16–5.38)	(2.28–5.20)	(2.18–5.56)	(2.44–4.78)
***p*-value**	**<0.01**		0.27		0.51		0.76		0.26	

Boldface values stand for significant effect (*p* < 0.05).

**Table 4:** Maternal dietary intakes (median and IQR) during late pregnancy and infant allergen sensitization and IgE-mediated food allergy outcomes.

	Allergen Sensitization			IgE-Mediated Food Allergy		
**Maternal Dietary Intakes**	Yes 165/629 (26%)	No 464/629 (74%)	*p* Value	Yes 91/632 (14%)	No 541/632 (86%)	*p* Value
**Energy**	7959	8094	0.94	8107	7975	0.75
	(6880–9713)	(6469–10,079)		(6854–9828)	(6528–9864)	
**Carbohydrate**	212.96	204.50	0.16	217.37	205.00	0.08
	(167.8–272.0)	(150.94–268.05)		(179.81–272.73)	(153.59–268.90)	
**Total fiber**	24.10	23.49	0.60	23.62	23.90	0.47
	(19.61–28.51)	(18.40–29.31)		(19.43–27.41)	(18.61–29.54)	
**Soluble fiber**	5.71	5.55	0.55	5.69	5.57	0.83
	(4.42–7.00)	(4.41–6.94)		(4.41–6.71)	(4.41–6.96)	
**Insoluble fiber**	7.06	6.59	0.30	7.08	6.60	0.18
	(5.19–8.66)	(4.99–8.58)		(5.61–8.89)	(5.60–8.61)	
**Prebiotic fiber**	1.41	1.50	0.63	1.40	1.50	0.31
	(1.06–1.93)	(1.07–1.96)		(1.01–1.86)	(1.09–1.96)	
**Resistant**	2.84	2.70	0.40	2.90	2.70	0.28
**starch**	(2.22–3.51)	(2.11–3.54)		(2.31–3.50)	(2.10–3.52)	
**Total fiber, grains**	9.44	9.20	0.78	9.70	9.20	0.38
	(6.96–12.08)	(7.00–11.63)		(7.23–12.26)	(6.91–11.70)	
**Total fiber, gluten rich**	7.40	7.70	0.73	7.40	7.70	0.46
	(5.71–10.92)	5.42–10.21		(5.91–11.20)	(5.41–10.21)	
**Total fiber, vegetables**	3.56	3.50	0.84	3.59	3.46	0.90
	(2.44–4.71)	(2.48–4.77)		(2.44–4.51)	(2.47–4.84)	
**Total fiber, green vegetables**	1.17	1.10	0.43	1.30	1.10	0.23
	(0.74–1.63)	(0.70–1.62)		(0.75–1.67)	(0.72–1.62)	
**Total fiber, fruit**	3.50	3.50	0.85	3.30	3.58	0.12
	(2.23–5.25	(2.22–5162)		(1.77–4.75)	(2.29–5.25)	

**Table 5:** Logistic regression analysis association between maternal dietary fiber intakes during late pregnancy and infant wheeze and eczema.

	Parent Reported Wheeze		Doctor Diagnosed Wheeze		Parent Reported Eczema		Doctor Diagnosed Eczema	
	OR (95% CI)	aOR (95% CI) **§**	OR (95% CI)	aOR (95% CI) **§**	OR (95% CI)	aOR (95% CI) **§**	OR (95% CI)	aOR (95% CI) **§**
**Carbohydrate**	0.99 (0.99, 1.00)	0.99 (0.99, 1.04)	1.00 (1.00, 1.00)	1.00 (0.99, 1.00)	1.00 (1.00, 1.00)	1.00 (1.00, 1.00)	1.00 (1.00, 1.00)	1.00 (1.00, 1.00)
***p* value**	0.39	0.32	0.32	0.11	0.10	0.14	**0.02**	0.05
**Total dietary fiber**	0.98 (0.96, 1.00)	0.99 (0.99, 1.00)	0.96 (0.93, 1.00)	0.98 (0.94, 1.01)	1.02 (1.01, 1.04)	1.03 (1.01, 1.05)	1.02 (0.99, 1.04)	1.01 (0.99, 1.04)
***p* value**	0.08	0.31	0.1	0.2	**0.04**	**0.04**	0.08	0.18
**Soluble fiber**	0.89 (0.82, 0.98)	0.91 (0.83, 1.00)	0.85 (0.73, 0.99)	0.85 (0.73, 1.00)	0.99 (0.93, 1.07)	0.98 (0.91, 1.07)	1.03 (0.95, 1.11)	1.01 (0.93, 1.10)
***p* value**	**0.02**	0.06	**0.04**	0.05	0.91	0.77	0.48	0.81
**Insoluble fiber**	0.96 (0.90, 1.02)	0.97 (0.92, 1.03)	1.00 (0.92, 1.09)	0.99 (0.90, 1.09)	0.95 (0.91, 0.99)	0.93 (0.89, 0.98)	0.99 (0.94, 1.04)	0.97 (0.92, 1.02)
***p* value**	0.17	0.35	0.85	0.94	**0.04**	**0.01**	0.71	0.25
**Resistant starch**	0.93 (0.79, 1.10)	0.96 (0.81, 1.15)	0.68 (0.50, 0.94)	0.68 (0.49, 0.95)	1.25 (1.08, 1.45)	1.27 (1.09, 1.49)	1.19 (1.02, 1.38)	1.19 (1.01,1.41)
***p* value**	0.39	0.67	**0.02**	**0.02**	**<0.01**	**<0.01**	**0.02**	**0.04**
**Prebiotic fiber**	0.94 (0.72, 1.23)	1.00 (0.75, 1.34)	0.88 (0.56, 1.38)	0.95 (0.60, 1.48)	1.05 (0.83, 1.33)	1.05 (0.82, 1.36)	1.04 (0.82, 1.33)	1.01 (0.78, 1.32)
***p* value**	0.65	0.97	0.58	0.82	0.63	0.68	0.74	0.93
**Fiber, grains**	0.97 (0.93, 1.02)	0.98 (0.93, 1.02)	0.94 (0.87, 1.01)	0.93 (0.86, 1.02)	1.01 (0.98, 1.05)	1.01 (0.97, 1.05)	1.03 (0.99, 1.08)	1.02 (0.98, 1.07)
***p* value**	0.18	0.36	0.12	0.09	0.47	0.69	0.07	0.25
**Fiber, gluten foods**	0.97 (0.94, 1.02)	0.98 (0.94,1.04)	0.95 (0.89, 1.03)	0.95 (0.88, 1.03)	1.03 (0.99, 1.07)	1.03 (0.99, 1.07)	1.05 (1.01, 1.09)	1.03 (0.99, 1.08)
***p* value**	0.34	0.63	0.24	0.25	0.09	0.16	**0.03**	0.1
**Fiber, green veg**	1.09 (0.87, 1.36)	1.08 (0.85, 1.36)	1.19 (0.84, 1.67)	1.09 (0.76, 1.55)	1.14 (0.93, 1.39)	1.18 (0.95, 1.46)	1.27 (1.03, 1.56)	1.32 (1.06, 1.64)
***p* value**	0.45	0.53	0.33	0.61	0.22	0.12	**0.02**	**0.01**
**Fiber, fruits**	0.89 (0.82, 0.97)	0.90 (0.83, 0.98)	0.93 (0.79, 1.11)	0.94 (0.83, 1.09)	1.00 (0.94, 1.07)	0.99 (0.93, 1.06)	0.99 (0.93, 1.07)	0.98 (0.92, 1.05)
***p* value**	**<0.01**	**0.02**	0.27	0.39	0.96	0.79	0.98	0.6

**§** adjusted odds ratio (aOR) after adjusting for confounders of infant gestational age, infant birth weight, maternal age, parity, ethnicity, education, delivery mode, gender of child, and pet ownership. Boldface values stand for significant effect (*p* < 0.05).
